# Closing the loop on EGFR therapy: decoding cetuximab response through circ-EGFR

**DOI:** 10.1038/s44321-025-00332-1

**Published:** 2025-11-10

**Authors:** Camilla Pilati, Pierre Laurent-Puig

**Affiliations:** 1 Centre de Recherche des Cordeliers, Inserm U1138, Sorbonne Université, Université Paris Cité. Personalized Medicine, Pharmacogenomics and Therapeutic Optimization. Equipe Labellisée Ligue Contre le Cancer, Paris, France; 2 Institut du Cancer Paris CARPEM, AP-HP, Department of Genetics and Molecular Medicine, Hôpital Européen Georges Pompidou, Paris, France

**Keywords:** Cancer, Digestive System, RNA Biology

## Abstract

P. Laurent-Puig and C. Pilati discuss the article by S. Sui et al, in this issue of *EMBO Mol Med*, that identifies circ-EGFR as a predictor of response to Cetuximab and a potential target in colorectal cancer.

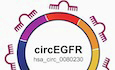

## Teaser

The introduction of monoclonal antibodies targeting the epidermal growth factor receptor (EGFR) has represented a milestone in the management of metastatic colorectal cancer (mCRC). Cetuximab significantly improves outcomes in patients with *RAS* wild-type tumors, yet only about one third derive durable benefit. This discrepancy underscores the biological heterogeneity of EGFR signaling and the urgent need for biomarkers capable of predicting therapeutic response beyond *RAS* status.

Over the past decade, several tumor-based predictors of cetuximab efficacy have been identified, yet most function as negative selectors. Mutations in *KRAS*, *NRAS*, and *BRAF* remain the most clinically validated markers, reliably identifying patients who will not benefit from EGFR blockade. Amplifications or activating alterations in *ERBB2* (HER2) and *MET* confer similar resistance, prompting the concept of “negative hyper-selection,” where additional genomic exclusions refine patient eligibility. Beyond mutational status, transcriptional and stromal determinants modulate cetuximab sensitivity. Expression of EGFR ligands AREG and EREG associates with benefit in *RAS* wild-type tumors (Appleyard et al, 2025). Transcriptomic stratification into Consensus Molecular Subtypes (CMS) also informs microenvironment-dependent drug response; CMS2 epithelial tumors are generally more responsive than CMS4 mesenchymal tumors characterized by TGF-β activation and stromal exclusion (Ten Hoorn et al, 2022). These studies highlight the complexity of cetuximab response, shaped by both tumor-intrinsic signaling and the surrounding stroma.

In parallel, liquid biopsy approaches have emerged as minimally invasive strategies to monitor treatment efficacy and clonal evolution. Circulating tumor DNA (ctDNA) enables dynamic monitoring of *RAS* mutations during anti-EGFR therapy, capturing on-treatment clonal selection and re-sensitization after drug withdrawal (Parseghian et al, 2019). Complementary prospective analyses confirmed that early ctDNA declines predict therapeutic efficacy in mCRC, supporting its integration as a real-time marker of drug response (Garlan et al, 2017).

Beyond circulating DNA, RNA-based biomarkers have also been explored as minimally invasive predictors of treatment response. Among them, microRNAs (miRNAs) have attracted particular interest given their regulatory roles in EGFR signaling and chemotherapy resistance. In metastatic CRC, a few circulating miRNAs—such as miR-345 in whole blood and miR-31-3p in tissue—have been associated with cetuximab efficacy, although none has achieved clinical validation (Schou et al, 2014; Laurent-Puig et al, 2019). Their limited stability in plasma, due to RNase-mediated degradation and variable carrier association, likely explains the difficulty of translating tissue biomarkers into robust liquid biopsy assays.

This limitation has shifted attention toward circular RNAs (circRNAs), covalently closed RNA loops generated by back-splicing of pre-mRNAs. This topology confers exceptional resistance to exonucleases and ensures long half-lives in plasma, exosomes, and even FFPE tissue. Functionally, circRNAs modulate transcriptional and post-transcriptional networks by sponging miRNAs, interacting with RNA-binding proteins (RBPs), altering splicing, or even encoding micro-peptides; their stability and detectability in biofluids position them as next-generation RNA biomarkers (Kristensen et al, 2022).

In colorectal cancer (CRC), a growing body of evidence links circRNAs to tumor progression, metabolic adaptation, and therapeutic resistance. Most dysregulated circRNAs are upregulated in tumor samples and promote oncogenic programs. High-throughput studies highlight recurrent oncogenic circRNAs in CRC, including circ-0053277, circVAPA, hsa_circ_0026416, circACAP2, and hsa_circ_0136666, which drive proliferation, invasion, and EMT while being detectable in plasma, supporting their potential as non-invasive biomarkers (Ameli-Mojarad et al, 2021; Nambidi et al, 2025).

Beyond growth control, circRNAs are increasingly implicated in treatment response. Across CRC cohorts and models, multiple circRNAs modulate chemosensitivity and resistance by tuning apoptosis, autophagy, EMT, and metabolic stress responses via miRNA–mRNA networks or interactions with RBPs. Notable CRC-linked examples include circRNAs associated with 5-fluorouracil (5-FU) and oxaliplatin response (e.g., circNRIP1, circACC1, hsa_circ_0076691), acting through axes that converge on AKT/mTOR signaling. Several of these transcripts are also detectable in circulation, underscoring the feasibility of liquid biopsy approaches for dynamic monitoring (Ameli-Mojarad et al, 2021; Nambidi et al, 2025).

Yet, despite this expanding literature, no circRNA had been clinically validated as a predictive biomarker of response to targeted therapy, leaving a gap in EGFR-directed treatment selection.

**Figure 1:**
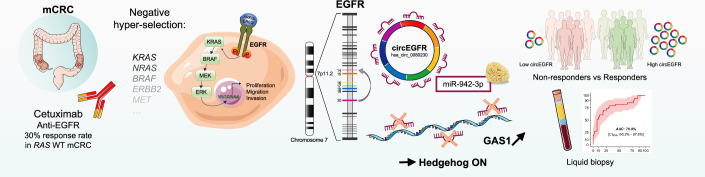
circ-EGFR links non-coding RNA biology to cetuximab response in mCRC. Only 30% of RAS wild-type mCRC patients benefit from cetuximab after “negative hyper-selection”. circ-EGFR (hsa_circ_0080230), derived from EGFR exons 14–20, sponges miR-942-3p and derepresses GAS1, activating Hedgehog signaling. High circ-EGFR levels in tissue or plasma identify responders to anti-EGFR therapy. This figure was created using elements from Servier Medical Art, which are freely available under a Creative Commons Attribution 3.0 Unported License.

In this issue of *EMBO Molecular Medicine*, Sui et al identify circular EGFR RNA (circ-EGFR) as a promising biomarker and potential therapeutic target in mCRC, marking the first demonstration of a circRNA with clinical utility for predicting cetuximab response (Sui et al, 2025) (Fig. 1). circ-EGFR expression was elevated in cetuximab-sensitive CRC cells and in responding patients. In a prospective cohort of 45 patients with *KRAS* wild-type mCRC, circ-EGFR levels stratified responders from non-responders with an AUC of 0.77; progression-free survival was nearly doubled among patients with high circ-EGFR expression. These findings were replicated using a plasma-based assay in an independent validation cohort of 112 patients, with a comparable AUC of 0.77, supporting the feasibility of non-invasive detection.

Mechanistic experiments reinforced these observations. circ-EGFR overexpression restored cetuximab sensitivity in resistant cells and xenograft models, whereas silencing enhanced resistance and invasiveness. Functionally, circ-EGFR acted as a sponge for miR-942-3p, thereby de-repressing GAS1 and activating Hedgehog signaling. This pathway sensitized tumor cells to EGFR blockade, and pharmacologic Hedgehog inhibition produced synergy with cetuximab in vitro. Together, these data position circ-EGFR not only as a biomarker of response but also as a functional mediator of sensitivity, bridging non-coding RNA biology, oncogenic signaling, and therapeutic efficacy.

Beyond its scientific novelty, the study opens translational opportunities. A plasma-based circ-EGFR test could refine patient selection, reducing unnecessary cetuximab exposure, potentially sparing a substantial fraction of *RAS* wild-type patients unlikely to benefit, while preserving efficacy for responders. The mechanistic link between circ-EGFR and Hedgehog signaling also motivates rational combinations, with preliminary data supporting synergy between cetuximab and Hedgehog inhibitors. Finally, the demonstration that circRNAs can be stably and reproducibly quantified from plasma underscores their broader potential as liquid biopsy biomarkers for therapy response, resistance monitoring, and relapse detection across cancer types.

While compelling, the findings are subject to technical limitations inherent to circRNA detection. Standard poly(A)-selected RNA-seq workflows are optimized for linear transcripts and largely exclude back-spliced reads, leading to systematic underrepresentation. Accurate profiling therefore requires rRNA-depleted or RNase R-treated libraries and back-splice–aware algorithms, which currently limit cross-study comparability and large-scale validation.

Despite these limitations, the convergence of mechanism, assay development, and clinical correlation provides a persuasive proof-of-concept. circ-EGFR exemplifies how non-coding RNAs can refine precision oncology in CRC: predicting who truly benefits from cetuximab and revealing new therapeutic vulnerabilities. This work marks an important step toward integrating circular RNAs into the armamentarium of predictive biomarkers as actionable tools for personalized cancer care.
